# Genome-Wide Analysis of the RAV Transcription Factor Genes in Rice Reveals Their Response Patterns to Hormones and Virus Infection

**DOI:** 10.3390/v13050752

**Published:** 2021-04-25

**Authors:** Changhai Chen, Yanjun Li, Hehong Zhang, Qiang Ma, Zhongyan Wei, Jianping Chen, Zongtao Sun

**Affiliations:** State Key Laboratory for Managing Biotic and Chemical Threats to the Quality and Safety of Agro-Products, Key Laboratory of Biotechnology in Plant Protection of Ministry of Agriculture and Zhejiang Province, Institute of Plant Virology, Ningbo University, Ningbo 315211, China; changhaichen@163.com (C.C.); liyanjun@nbu.edu.cn (Y.L.); 18758878079@163.com (H.Z.); maq1325356994@163.com (Q.M.); w_zhongyan@163.com (Z.W.)

**Keywords:** genome-wide analysis, RAV transcription factor, hormone treatment, virus infection, rice

## Abstract

The RAV family is part of the B3 superfamily and is one of the most abundant transcription factor families in plants. Members have highly conserved B3 or AP2 DNA binding domains. Although the *RAV* family genes of several species have been systematically identified from genome-wide studies, there has been no comprehensive study to identify rice *RAV* family genes. Here, we identified 15 genes of the RAV family in the rice genome and analyzed their phylogenetic relationships, gene structure, conserved domains, and chromosomal distribution. Based on domain similarity and phylogenetic topology, rice RAV transcription factors were phylogenetically clustered into four groups. qRT-PCR analyses showed that expression of these *RAV* genes was significantly up-regulated or down-regulated by plant hormone treatments, including BL, NAA, IAA, MeJA, and SA. Most of the rice *RAV* genes were dramatically down-regulated in response to rice stripe virus (RSV) and mostly up-regulated in response to Southern rice black-streaked dwarf virus (SRBSDV). These results suggest that the rice *RAV* genes are involved in diverse signaling pathways and in varied responses to virus infection.

## 1. Introduction

Plants are regulated by the coordinated expression of thousands of genes throughout growth and development. Transcription factors (TFs) play a key role in these processes by recognizing certain nucleotide sequences (motifs) before or after genes (upstream and downstream) on chromosomes to self-regulate or regulate the transcription of downstream target genes. TFs usually contain a DNA binding domain (DBD), a transcription activation domain, a nuclear localization signal, and an oligomerization site [1]. These domains work together to mediate many physiological and biochemical processes. At the same time, TFs can activate and/or inhibit transcription when subjected to endogenous and exogenous stimulation [2,3]. Most TFs belong to one of a number of gene families, making their regulation complex and orderly [2].

The RELATED TO ABI3/VP1 (RAV) family is part of the B3 superfamily, which contains plant-specific transcription factors including the AUXIN RESPONSE FACTOR (ARF), LEAFY COTYLEDON2 (LEC2)–ABSCISIC ACID INSENSITIVE3 (ABI3)–VAL (LAV), and REPRODUCTIVE MERISTEM ARF (REM) families [4]. Many of the B3 genes in the ARF and LAV families have been studied in detail, but B3 genes in the RAV family are less well understood. The RAV family proteins contain a B3 domain and/or an AP2 domain. Therefore, members of the RAV family can be reasonably classified as members of the B3 superfamily or AP2/EREBP family [4,5]. The B3 domain, which consists of about 110 amino acids, is a DNA binding domain named because of its position in the third basic domain of the maize gene *VIVIPAROUS1* (*VP1*) [6]. The AP2 domain is also a DNA binding domain that was first identified in the AP2 protein of *Arabidopsis* (*Arabidopsis thaliana*) [7].

RAV transcription factors have documented functions in several aspects of plant growth and development. For example, it was reported that *Arabidopsis* plants ectopically expressing *RAV1* exhibited fewer lateral roots and rosette leaves, and bloomed later [8]. Plants in which *RAV1* was co-suppressed had normal roots but flowered earlier than the wild type [8]. Similar phenotypes were observed when soybean *RAV1* homologous genes were overexpressed in tobacco [9]. These results indicate that *RAV1* is a negative regulator of plant growth and flowering. *NGATHA* genes (*NGA1–NGA4*), which are *RAV* genes lacking the AP2 domain [10], also play important roles in leaf and flower development. *nga1*/*nga2*/*nga3*/*nga4* quadruple mutants showed leaf shape defects and several flower defects [11]. In addition to the B3 domain, RAV family members *TEM1* and *TEM2*, which contain the AP2 domain, are negative regulators of flowering [11] by repressing the expression levels of *FLOWERING LOCUS T* (*FT*) and gibberellins [11]. Besides, Osnato et al. first identified and named 12 *RAV* genes (*OsRAV1–OsRAV12*) in rice and also found that *OsRAV9*/*OsTEM1* inhibits photoperiod flowering upstream of the flower activators *OsMADS14* and *Hd3a* [12]. In addition, *OsRAV11* and *OsRAV12*, which act downstream of the flower homeotic factor, may have acquired a new function in carpel differentiation and seed size control [12]. These results fully demonstrate the important roles of the B3 gene in the regulation of plant growth and development.

RAV transcription factors are also known to play roles in regulation of plant responses to biotic and abiotic stresses, including plant pathogens and hormones. Previous studies have reported that cassava RAV transcription factors *MeRAV1* and *MeRAV2* are essential for disease resistance against cassava bacterial blight by activating melatonin biosynthesis genes [13]. In *Arabidopsis thaliana*, overexpression of the pepper *CARAV1* gene induced the expression of some pathogenesis-related genes, conferring resistance to *Pseudomonas syringae pv. tomato* DC3000 [14,15]. Furthermore, RAV family members can be affected by various plant hormones and are known to be involved in ethylene and brassinosteroid responses [8,16]. *Arabidopsis RAV1* was down-regulated by 24-epibrassinolide treatment [8], and transgenic *Arabidopsis* plants overexpressing *RAV1* were insensitive to ABA [17]. It is clear, therefore, that *RAV* genes are important in development and response to abiotic/biotic stresses in various different plants.

There have been genome-wide analyses of *RAV* family genes in several plant species, but little is known about the RAV family in rice. Rice (*Oryza sativa*) is an edible starchy cereal and grass plant (family Poaceae). Roughly one-half of the world population is wholly dependent upon rice as a staple food [18]. Like other crops, rice growth and productivity are greatly affected by various biotic and abiotic stresses, and plants activate resistance to these stresses by first inducing or inhibiting the expression of transcription factors. Given the potential importance of RAV TFs in plant responses to abiotic/biotic stresses, the aim of the study is to identify, classify, and characterize the roles of rice *RAV* genes. In this study, we carried out a genome-wide analysis of the rice *RAV* gene family and investigated the potential functions of *RAV* genes in rice responses to various hormone treatments and virus infection.

## 2. Materials and Methods

### 2.1. Searches for RAV Genes in the Rice, Arabidopsis, and Maize Genome Databases

The whole genome sequences and predicted RAV protein sequences of rice, *Arabidopsis*, and maize were obtained from Phytozome’s Osativa Genome 7.0 version, TAIR, and ensemble plant Zea_mays.B73_RefGen_v4.pep version, respectively [19].

To find possible RAV family members in rice and maize, OsRAV15 and Zm00001d043782_T001 [20] were used as protein queries, respectively, and BLAST Compare Two Seqs of TBtools tools [21] were used. Candidate genes were identified by BLASTP using distance score value ≥ 100 and an e-value ≤ 1 × 10^−10^, using NCBI Conserved Domain Search Service (CD search) [22,23,24]. To exclude duplicate sequences, ClustalX was used to align all candidate RAV sequences, which were then manually checked. After manually filtering out the duplicate sequences, a total of 15 rice *RAV*s and 15 maize *RAV*s were obtained. To investigate the protein properties of the putative RAV proteins, their theoretical isoelectric points (pI) were calculated using the calculation pI tool in the ExPASy server [25,26].

### 2.2. Multiple Sequence Alignment and Phylogenetic Analysis

Phylogenetic trees were produced using the full-length amino acid sequences of RAV proteins from rice, *Arabidopsis*, and maize, which were aligned using Clustal X 2.1 [27,28]. Phylogenetic analyses were carried out using MEGAX by the Maximum-likelihood (ML) and Neighbor-Joining (NJ) methods and with 1000 bootstrap replicates [29,30].

### 2.3. Gene Structure and Conserved Motif Analysis

The exon–intron substructure map of each *RAV* gene was generated using Tools Online GSDS [31,32]. The genome annotation file (protein) for *Osativa* was acquired from phytozome [19,33]. Motif analysis was carried out using TBtools Visualize Domain Pattern (from NCBI Batch-CDD) and NCBI Conserved Domain Search Service (CD search) [21,22,23].

### 2.4. Chromosomal Distribution of RAV Genes

The chromosome distribution information of the identified genes was searched against the rice group database. The position of each RAV was marked on the chromosome using Perl script.

### 2.5. Plant Materials and Growth Conditions

Rice seeds of cultivars nanjing9108 and Nip were used as plant materials in this study. Rice plants were grown in a greenhouse with a 14/10 h light/dark cycle at 28–30 °C.

### 2.6. Hormone Treatments

Stock solutions of Epibrassinolide (BL, Sigma-Aldrich, St. Louis, MO, USA), methyl jasmonate (MeJA, TCI, Tokyo, Japen), 1-naphthylacetic acid (NAA, Sigma-Aldrich, St. Louis, MO, USA), indole-3-acetic acid (IAA, Sigma-Aldrich, St. Louis, MO, USA), and salicylic acid (SA, Sigma-Aldrich, St. Louis, MO, USA) were prepared in 100% ethanol and diluted to the appropriate concentration with sterile distilled water containing 0.1% Triton X-100 for experiments. The same volume of ethanol with 0.1% Triton X-100 was used as a mock control. To evaluate the effects of BL, NAA, IAA, MeJA, and SA on *RAVs*, rice seedlings were sprayed with 10 μmol BL, 5 μmol NAA, 5 μmol IAA, 100 μmol MeJA, and 500 μmol SA, respectively. Eighty seedlings per hormonal treatment were sprayed, and samples were collected for testing at 3, 6, and 12 h.

### 2.7. RSV and SRBSDV Infection

RSV and SRBSDV were inoculated to rice seedlings at the 2 to 3 leaf stage as described previously [34,35]. In short, each treatment had three biological replicates, and each replicate was inoculated with SBPH containing RSV or WBPH containing SRBSDV. Three viruliferous nymphs were fed on each plant for 3.5 days and were then completely removed. The inoculated plants were grown in the greenhouse and checked for the development of symptoms.

### 2.8. RNA Extraction and qRT-PCR

In order to analyze gene expression levels, total RNA was isolated from hormone-treated rice leaves and RSV- and SRBSDV-infected leaves using TRIzol reagent (Invitrogen, Carlsbad, CA, USA) according to the manufacturer’s protocol. First-strand cDNA was synthesized with Tiangen Rapid Quantitative RT Kit with gDNase (Tiangen, Beijing, China). qRT-PCR analysis was performed using the ABI QuantStudio 5 sequence detection system (Applied Biosystems, Thermo Fisher, Waltham, MA, USA) and ChamQTM SYBR qPCR Master Mix (Low ROX Premixed, Vazyme, Nanjing, China). *OsUBQ5* (AK061988) was used as an internal control [36]. The relative expression levels of genes were determined using the 2^−ΔΔ*C*t^ method [37]. Each treatment had three biological replicates, each biological replicate had three to five plants, and each sample had three technical replicates. The primers used for qRT-PCR are listed in Table S1.

### 2.9. Statistical Analysis

One-way or two-way analysis of variance and Fisher’s least significant difference were used for data analysis, and *p* value ≤ 0.05 was considered statistically significant. All analyses were performed using OriginPro 9.1 software [38].

## 3. Results

### 3.1. Identification and Phylogenetic Analysis of RAV Transcription Factors in Rice

By BLAST Compare Two Seqs of TBtools tools, NCBI CD search, and comparisons with known rice RAV family proteins, fifteen non-redundant rice *RAV* genes were identified, including 12 known rice *RAV* genes, and were named *OsRAV1* to *OsRAV15* (Table 1). Besides, in order to compare RAV family proteins between monocotyledonous plants, fifteen non-redundant maize *RAV* genes were also identified using the same method (Table S2).

**Figure 1:**
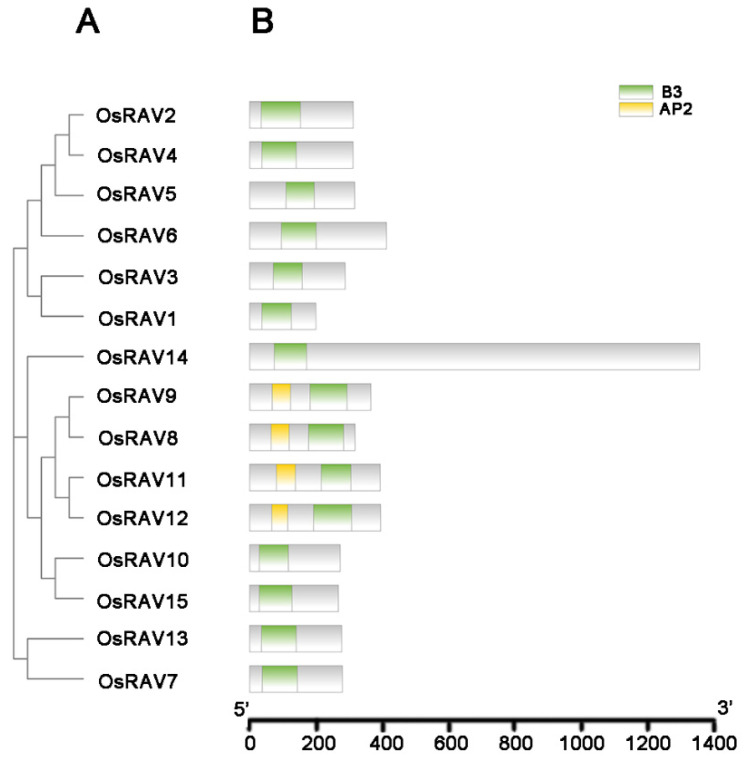
Phylogenetic relationship of RAV family proteins in rice. The phylogenetic tree was produced using MEGA-X software based on the comparison of amino acids of the RAV proteins. The neighbor-joining method was used with 1000 bootstrap replicates. (**A**) Neighbor-joining phylogenetic tree of the RAV family in Os (*Oryza sativa*). (**B**) Conserved motif distributions of the RAV proteins in rice. The B3 domains and AP2 domains are indicated by the green and yellow boxes, respectively.

**Figure 2:**
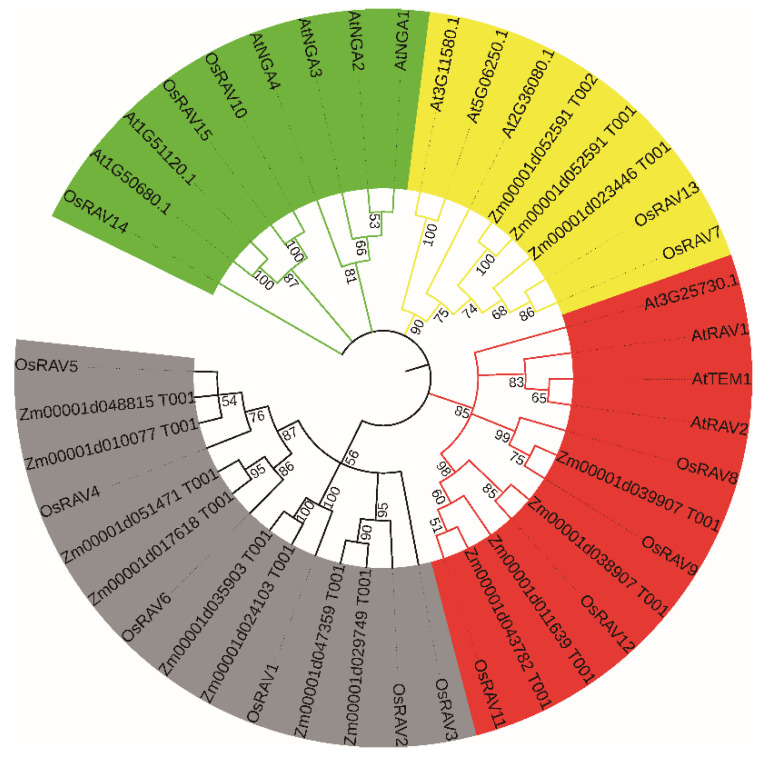
Phylogenetic relationship of the RAV families of At (*Arabidopsis thaliana*), Os (*Oryza sativa*), and maize (*Zea mays*).The four colors represent four different groups of the RAV family.

In a neighbor-joining phylogenetic analysis of the 15 rice RAVs, 13 *Arabidopsis*, and 15 maize RAV proteins, the *RAV* genes were divided into four groups (Figure 1 and Figure 2). Of the 15 rice *RAV* genes, *OsRAV8*, *OsRAV9*, *OsRAV11*, and *OsRAV12* belong to the same group and contain both B3 and AP2 domains (Figure 2). Similarly, 6 of the 13 *Arabidopsis* RAV proteins contain the AP2 domain, and 4 of the 15 maize RAV proteins contain the AP2 domain [4,19]. The other members of the rice RAV family have only a B3 domain. The group shown in red in Figure 2 contains the AP2 domain, which has been reported to be associated with flowering time in both *Arabidopsis* and rice. The group shown in black has been reported to be involved in flower and leaf development.

### 3.2. Gene Structure and Domain Analysis of Rice RAV Genes

**Figure 3:**
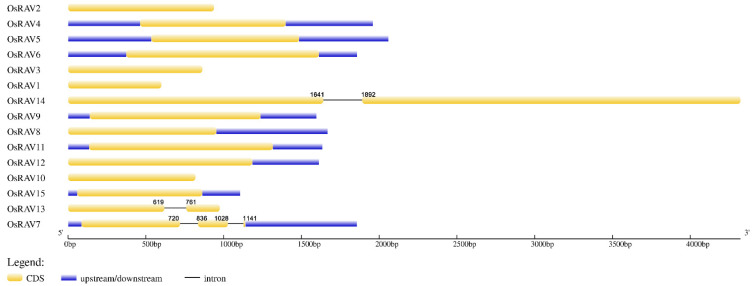
The exon–intron structure of the rice *RAV* genes. The exon–intron structures were generated using the GSDS online tool. The exons, introns, and untranslated regions (UTRs) are represented by yellow boxes, black lines, and blue boxes, respectively.

The structural diversity of the rice *RAV* genes is shown in Figure 3, which displays the exon–intron structure, and in Figure 1B and Figure S1, which show the conserved domains for each gene. Of the 15 genes, only 3 contained introns: *OsRAV7* (2 introns), *OsRAV13* (1 intron), and *OsRAV14* (1 intron). The introns of *OsRAV7* and *OsRAV13* were towards the 3’ end of the gene, while the only intron of *OsRAV14* was located in the middle region of the gene. The ORF lengths ranged from 600 to 4074 bp, encoding proteins of 199 to 1357 amino acids (aa) and with isoelectric point (pI) values between 5.03 and 9.80 (Table 1).

### 3.3. Chromosomal Distribution Analysis of Rice RAV Genes

**Figure 4:**
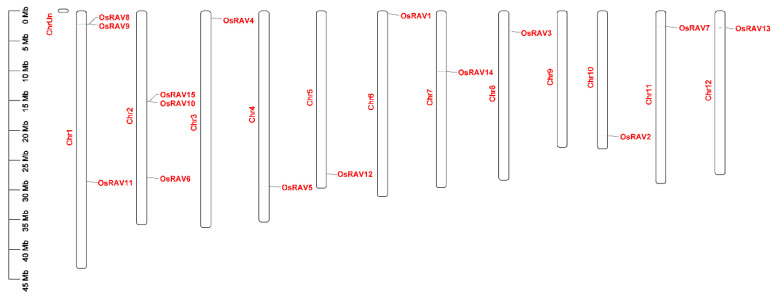
Distribution of *RAV* genes on the 12 chromosomes of Os (*Oryza sativa*). The *RAV* genes are shown on the right side of each chromosome. The gene location and the size of each chromosome can be estimated using the scale on the left side of the figure.

Genome chromosomal location analysis showed that the rice *RAV* genes were distributed among all of the 12 chromosomes except chromosome 9 (Chr9) (Figure 4). Chr1 and Chr2 each had three *RAV* genes, while each of the remaining chromosomes contained one *RAV* gene. *RAV* genes were found at the top of Chr1, Chr3, Chr6, Chr7, Chr8, Chr11, and Chr12, and the bottom of Chr1, Chr2, Chr4, Chr5, and Chr10. Except for Chr2, *RAVs* were not found in the central chromosome regions.

### 3.4. Expression Patterns of Rice RAV Genes under Hormone Treatments

**Figure 5:**
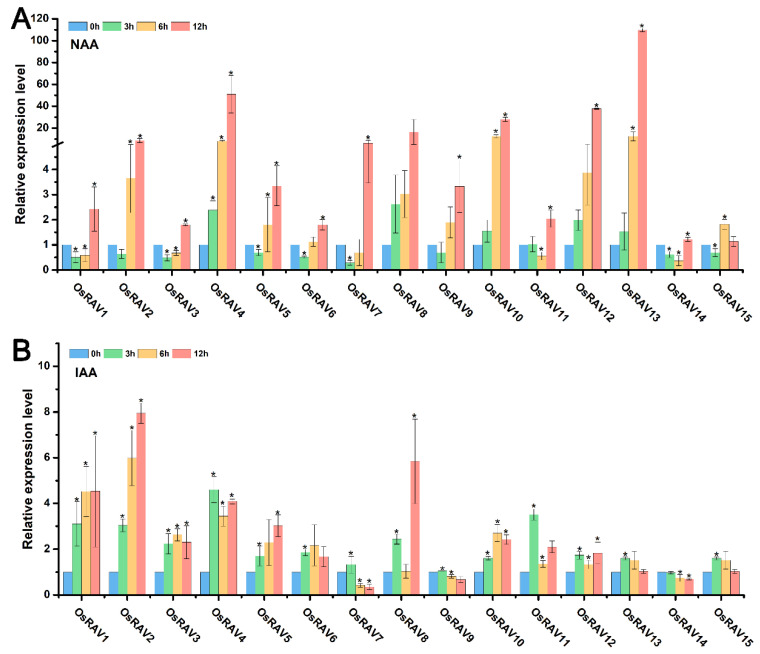
Relative expression level of fifteen *RAV* genes following hormone treatments. Plants were treated with NAA (50 μM) (**A**), IAA (50 μM) (**B**) and sampled at 0, 3, 6, and 12 h. qPCR data were normalized using *OsUBQ5*. Results are the means ± standard deviation (SD) of three biological replicates. * indicates significant difference from the mock control at *p* ≤ 0.05 by Fisher’s least significant difference tests.

**Figure 6:**
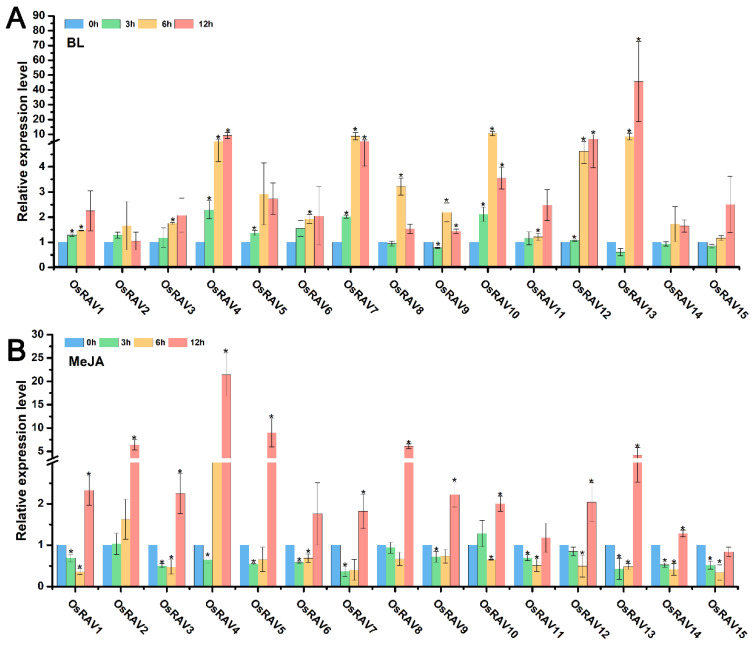
Relative expression level of fifteen *RAV* genes following hormone treatments. Plants were treated with BL (10 μM) (**A**), MeJA (100 μM) (**B**) and sampled at 0, 3, 6, and 12 h. qPCR data were normalized using *OsUBQ5*. Results are the means ± standard deviation (SD) of three biological replicates. * indicates significant difference from the mock control at *p* ≤ 0.05 by Fisher’s least significant difference tests.

**Figure 7:**
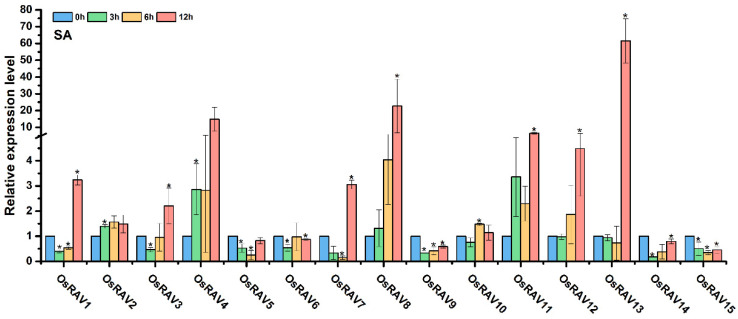
Relative expression level of fifteen *RAV* genes following SA (500 μM) treatments and sampled at 0, 3, 6, and 12 h. qPCR data were normalized using *OsUBQ5*. Results are the means ± standard deviation (SD) of three biological replicates. * indicates significant difference from the mock control at *p* ≤ 0.05 by Fisher’s least significant difference tests.

RAV proteins have well-characterized roles in plant physiology and development, and are known to be regulated and affected by many plant hormones. The response of rice *RAV* genes to hormone treatment was therefore examined using qRT-PCR to analyze the expression patterns of rice *RAV* genes under different hormone treatments, including Epibrassinolide (BL), 1-naphthylacetic acid (NAA), indole-3-acetic acid (IAA), methyl jasmonate (MeJA) and salicylic acid (SA). Most of the rice *RAV* genes were up-regulated by all five hormones at 12 h after treatment, but *OsRAV9*, *OsRAV14*, and *OsRAV15* were down-regulated by IAA and SA at this time point (Figure 5, Figure 6 and Figure 7). BL, NAA, and SA tended to induce higher expression of *RAV* genes than IAA and MeJA, especially in *OsRAV12* and *OsRAV13*. In response to NAA, MeJA, and SA, the expression of rice *RAV* genes generally decreased at 3 h in the early treatment period, and then increased steadily with time. Remarkably, 13 of the 15 rice *RAV* genes were down-regulated first and then up-regulated under MeJA treatment. This initial down-regulation was not observed following BL or IAA treatment. The results suggest that rice *RAV* genes are significantly involved in hormone response, but with different response patterns to different hormones.

### 3.5. Expression Profiling of Rice RAV Genes in Response to Rice Stripe Virus (RSV) and Southern Rice Black-Streaked Dwarf Virus (SRBSDV) Infection

**Figure 8:**
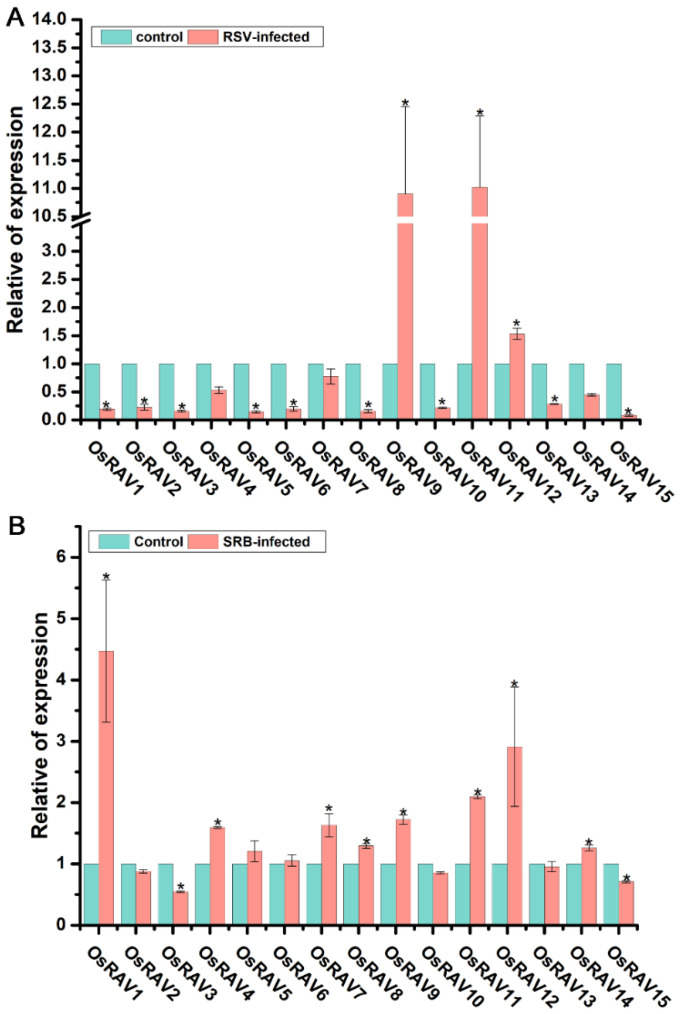
Relative expression level of fifteen *RAV* genes in response to RSV (**A**) and SRBSDV (**B**) infection. RNA was extracted from RSV-infected plants at 30 dpi and from SRBSDV-infected plants at 90 dpi. qPCR data are shown as relative expression levels of virus-infected plants in comparison with the mock control plants and normalized using *OsUBQ5*. Results are the means ± standard deviation (SD) of three biological replicates. * indicates significant difference between virus free and infected at *p* ≤ 0.05 by Fisher’s least significant difference tests.

RSV is a member of the genus *Tenuivirus* [39]. It is transmitted by the small brown planthopper (SBPH), *Laodelphax striatellus*, and causes large yield reductions in rice [40,41,42]. SRBSDV is a member of the genus *Fijivirus* (family *Reoviridae*) and is efficiently transmitted by the white-backed planthopper (WBPH), *Sogatella furcifera* [43]. In order to further investigate whether rice *RAV* genes participate in virus infection, the expression levels of rice *RAV* genes were analyzed in RSV- and SRBSDV-infected rice. qRT-PCR assays showed that the expression levels of most rice *RAV* genes, except *OsRAV9*, *OsRAV11*, and *OsRAV12*, were dramatically reduced in RSV-infected rice plants compared to mock control plants at 30 days post inoculation (dpi) (Figure 8A). In contrast, *OsRAV9*, *OsRAV11*, and *OsRAV12* were significantly up-regulated under RSV virus infection. These results implied that most of the *RAV* genes, except *OsRAV9*, *OsRAV11*, and *OsRAV12*, were repressed by RSV virus infection. By contrast, most *RAV* genes were up-regulated in response to SRBSDV. Thus, the expression levels of *OsRAV1*, *OsRAV3*, *OsRAV7*, *OsRAV8*, *OsRAV9*, *OsRAV11*, *OsRAV12*, and *OsRAV14* were all significantly increased in SRBSDV-infected rice plants compared to mock control plants (Figure 8B). *OsRAV9*, *OsRAV11*, and *OsRAV12* were therefore significantly up-regulated by both viruses. In contrast, *OsRAV3* and *OsRAV15* were both significantly inhibited after infection by either virus. The results suggest that rice *RAV* genes are significantly involved in RSV and SRBSDV infection.

## 4. Discussion

As one of the largest gene families in plants, the RAV family plays vital roles in a variety of physiological and biochemical processes by regulating the expression of genes involved in various stress conditions, as shown by studies in *Arabidopsis* [4], maize [20], and soybean [44]. However, there have been few studies of the structure and regulatory functions of *RAV* genes in rice. In this study, we systematically analyzed the phylogenetic relationships, gene structure, conserved domains, and chromosomal distribution of RAV family transcription factors in rice, and also analyzed the expression of RAV family transcription factors under different hormone treatments as well as RSV and SRBSDV virus infection.

The genome-wide analysis of the *RAV* family showed that there were 15 *RAV* genes in rice and 15 *RAV* genes in maize, compared with the 13 reported from *Arabidopsis* [4] and 13 in soybean [44]. Rice RAV transcription factors were phylogenetically clustered into four groups. Among these groups, most of the members in the red group contained both AP2 and B3 domains, and the members of the red group shared the same function in the flowering of both *Arabidopsis* and rice [11,12]. However, only 20% of the detected rice *RAV* genes contain introns. The loss of the introns in the gene is faster than the gain of the introns after segment repetition [45]. It has been reported that introns not only play a role in gene expression, but also participate in the evolution of genes, during which some genes will be lost and some will be retained [46,47]. Therefore, in the evolution of higher plants, the introns may have been lost from the *RAV* family genes. Genetic structural analysis showed that only 3 of the 15 *RAV* genes had introns, and whether the absence and retention of these introns affect the expression of *RAV* family genes remains to be further studied.

Previously studied RAV transcription factors function in the regulation of plant responses to biotic and abiotic stresses, including plant pathogens and hormones. In this study, the rice *RAV* genes were mostly up-regulated by five hormones when tested at 12 h after treatment, and all were significantly affected in some way. The overall expression trends were similar in response to NAA, MeJA, and SA, but differed from those of BL and IAA. This suggests that rice *RAV* genes, like those of other plants, are significantly involved in hormone response. However, there are different response patterns to different hormones, suggesting functional diversity among the genes. The exact mechanisms by which rice *RAV* genes participate in hormonal responses and exhibit different expression patterns needs to be further investigated.

Our results also demonstrate that rice *RAV* genes respond to virus infection, but while most were dramatically down-regulated after RSV infection, they were mostly up-regulated in response to SRBSDV. However, *OsRAV9*, *OsRAV11*, and *OsRAV12* were significantly up-regulated by both viruses, while *OsRAV3* and *OsRAV15* were down-regulated by both. It is clear that rice *RAV* genes have significant, but sometimes differing, roles in response to RSV and SRBSDV, and that *RAV* family genes are important both in hormone-mediated morphology such as cellular aging, and in disease resistance [13]. *RAV* family genes are functionally diverse and are indispensable in the normal development of rice. How these genes participate in these processes will require further investigation.

## Figures and Tables

**Table 1:** Properties of the rice RAV proteins identified, including sequence ID and predicted sequence length.

Number	Gene Name	Gene ID Number	Amino Acid Residues	B3 Domain	AP2 Domain	pI
1	*OsRAV1*	>LOC_Os06g01860	199	37–126		9.56
2	*OsRAV2*	>LOC_Os10g39190	312	35–153		9.62
3	*OsRAV3*	>LOC_Os08g06120	287	71–159		5.03
4	*OsRAV4*	>LOC_Os03g02900	311	37–141		9.80
5	*OsRAV5*	>LOC_Os04g49230	316	110–195		8.81
6	*OsRAV6*	>LOC_Os02g45850	412	96–201		7.33
7	*OsRAV7*	>LOC_Os11g05740	279	38–144		6.67
8	*OsRAV8*	>LOC_Os01g04750	317	176–271	65–119	7.08
9	*OsRAV9*	>LOC_Os01g04800	365	182–294	68–123	9.82
10	*OsRAV10*	>LOC_Os02g25830	272	28–132		6.85
11	*OsRAV11*	>LOC_Os01g49830	393	216–312	81–138	9.09
12	*OsRAV12*	>LOC_Os05g47650	394	193–308	67–123	9.51
13	*OsRAV13*	>LOC_Os12g06080	277	36–141		7.25
14	*OsRAV14*	>LOC_Os07g17230	1357	74–172		5.76
15	*OsRAV15*	>LOC_Os02g25820	267	28–128		9.02

## Data Availability

All of the materials and data that were used or generated in this study are described and available in the manuscript and supplementary files.

## Supplementary Materials

The following are available online at https://www.mdpi.com/article/10.3390/v13050752/s1, Figure S1: architecture of conserved protein motifs in RAV genes from rice, Table S1: list of primers used in qRT-PCR to determine relative expression levels, Table S2: properties of the maize RAV proteins identified, including sequence ID and predicted sequence length.

## Abbreviations

qRT-PCR	Quantitative real-time PCR
RSV	Rice stripe virus
SRBSDV	Southern rice black-streaked dwarf virus
TFs	Transcription factors
DBD	DNA binding domain
RAV	Related to ABI3/VP1
ARF	AUXIN RESPONSE FACTOR
LEC2	LEAFY COTYLEDON2
ABI3	ABSCISIC ACID INSENSITIVE3
REM	Reproductive meristem
VP1	VIVIPAROUS1
FT	Flowering locus T
ML	Maximum-likelihood
NJ	Neighbor-joining
BL	Epibrassinolide
MeJA	Methyl jasmonate
NAA	1-naphthylacetic acid
IAA	Indole-3-acetic acid
SA	Salicylic acid
Os	Oryza sativa
UTRs	Untranslated regions
SBPH	The small brown planthopper
WBPH	The white-backed planthopper
dpi	Days post inoculation

## References

[1] Ptashne M. (1988). How eukaryotic transcriptional activators work. Nature.

[2] Riechmann J.L., Heard J., Martin G., Reuber L., Jiang C.-Z., Keddie J., Adam L., Pineda O., Ratcliffe O.J., Samaha R.R. (2000). Arabidopsis Transcription Factors: Genome-Wide Comparative Analysis Among Eukaryotes. Science.

[3] Xiong Y., Liu T., Tian C., Sun S., Li J., Chen M. (2005). Transcription Factors in Rice: A Genome-wide Comparative Analysis between Monocots and Eudicots. Plant Mol. Biol..

[4] Swaminathan K., Peterson K., Jack T. (2008). The plant B3 superfamily. Trends Plant Sci..

[5] Matías-Hernández L., Aguilar-Jaramillo A.E., Marín-González E., Suárez-López P., Pelaz S. (2014). RAV genes: Regulation of floral induction and beyond. Ann. Bot..

[6] Kagaya Y., Ohmiya K., Hattori T. (1999). RAV1, a novel DNA-binding protein, binds to bipartite recognition sequence through two distinct DNA-binding domains uniquely found in higher plants. Nucleic Acids Res..

[7] Jofuku K.D., den Boer B.G., Van Montagu M., Okamuro J.K. (1994). Control of *Arabidopsis* flower and seed development by the homeotic gene APETALA2. Plant Cell.

[8] Hu Y.X., Wang Y.H., Liu X.F., Li J.Y. (2004). Arabidopsis RAV1 is down-regulated by brassinosteroid and may act as a negative regulator during plant development. Cell Res..

[9] Zhao L., Luo Q., Yang C., Han Y., Li W. (2008). A RAV-like transcription factor controls photosynthesis and senescence in soybean. Planta.

[10] Alvarez J.P., Pekker I., Goldshmidt A., Blum E., Amsellem Z., Eshed Y. (2006). Endogenous and Synthetic MicroRNAs Stimulate Simultaneous, Efficient, and Localized Regulation of Multiple Targets in Diverse Species. Plant Cell.

[11] Castillejo C., Pelaz S. (2008). The Balance between CONSTANS and TEMPRANILLO Activities Determines FT Expression to Trigger Flowering. Curr. Biol..

[12] Osnato M., Matias-Hernandez L., Aguilar-Jaramillo A.E., Kater M.M., Pelaz S. (2020). Genes of the RAV Family Control Heading Date and Carpel Development in Rice. Plant Physiol..

[13] Wei Y., Chang Y., Zeng H., Liu G., He C., Shi H. (2017). RAV transcription factors are essential for disease resistance against cassava bacterial blight via activation of melatonin biosynthesis genes. J. Pineal Res..

[14] Hong J.K., Lee S.C., Hwang B.K. (2005). Activation of pepper basic PR-1 gene promoter during defense signaling to pathogen, abiotic and environmental stresses. Gene.

[15] Sohn K.H., Lee S.C., Jung H.W., Hong J.K., Hwang B.K. (2006). Expression and functional roles of the pepper pathogen-induced transcription factor RAV1 in bacterial disease resistance, and drought and salt stress tolerance. Plant Mol. Biol..

[16] Alonso J.M., Stepanova A.N., Leisse T.J., Kim C.J., Chen H., Shinn P., Stevenson D.K., Zimmerman J., Barajas P., Cheuk R. (2003). Genome-Wide Insertional Mutagenesis of *Arabidopsis thaliana*. Science.

[17] Fu M., Kang H.K., Son S.-H., Kim S.-K., Nam K.H. (2014). A Subset of Arabidopsis RAV Transcription Factors Modulates Drought and Salt Stress Responses Independent of ABA. Plant Cell Physiol..

[18] Zhu L.-J., Liu Q.-Q., Wilson J.D., Gu M.-H., Shi Y.-C. (2011). Digestibility and physicochemical properties of rice (*Oryza sativa* L.) flours and starches differing in amylose content. Carbohydr. Polym..

[19] Phytozome, the Plant Comparative Genomics Portal of the Department of Energy’s Joint Genome Institute. https://phytozome.jgi.doe.gov/pz/portal.html.

[20] Du H., Huang M., Zhang Z., Cheng S. (2014). Genome-wide analysis of the AP2/ERF gene family in maize waterlogging stress response. Euphytica.

[21] Chen C., Chen H., Zhang Y., Thomas H.R., Frank M.H., He Y., Xia R. (2020). TBtools: An Integrative Toolkit Developed for Interactive Analyses of Big Biological Data. Mol. Plant.

[22] Lu S., Wang J., Chitsaz F., Derbyshire M.K., Geer R.C., Gonzales N.R., Gwadz M., I Hurwitz D., Marchler G.H., Song J.S. (2020). CDD/SPARCLE: The conserved domain database in 2020. Nucleic Acids Res..

[23] Marchler-Bauer A., Bo Y., Han L., He J., Lanczycki C.J., Lu S., Chitsaz F., Derbyshire M.K., Geer R.C., Gonzales N.R. (2017). CDD/SPARCLE: Functional classification of proteins via subfamily domain architectures. Nucleic Acids Res..

[24] Batch Web CD-Search Tool. https://www.ncbi.nlm.nih.gov/Structure/bwrpsb/bwrpsb.cgi.

[25] Wilkins M.R., Gasteiger E., Bairoch A., Sanchez J.-C., Williams K.L., Appel R.D., Hochstrasser D.F. (1999). Protein Identification and Analysis Tools in the ExPASy Server. Methods Mol. Biol..

[26] Bioinformatics Resource Portal of the SIB Swiss Institute of Bioinformatics Home. http://www.expasy.org/.

[27] Sievers F., Higgins D.G. (2018). Clustal Omega for making accurate alignments of many protein sequences. Protein Sci..

[28] Clustal: Multiple Sequence Alignment Home Page. http://www.clustal.org/.

[29] Kumar S., Stecher G., Li M., Knyaz C., Tamura K. (2018). MEGA X: Molecular evolutionary genetics analysis across computing platforms. Mol. Biol. Evol..

[30] Molecular Evolutionary Genetics Analysis Home Page. http://www.megasoftware.net/.

[31] Hu B., Jin J., Guo A.-Y., Zhang H., Luo J., Gao G. (2015). GSDS 2.0: An upgraded gene feature visualization server. Bioinformatics.

[32] GSDS 2.0: Gene Structure Display Server Home Page. http://gsds.gao-lab.org/.

[33] Ouyang S., Zhu W., Hamilton J., Lin H., Campbell M., Childs K., Thibaud-Nissen F., Malek R.L., Lee Y., Zheng L. (2006). The TIGR Rice Genome Annotation Resource: Improvements and new features. Nucleic Acids Res..

[34] Zhang H., Li L., He Y., Qin Q., Chen C., Wei Z., Tan X., Xie K., Zhang R., Hong G. (2020). Distinct modes of manipulation of rice auxin response factor OsARF17 by different plant RNA viruses for infection. Proc. Natl. Acad. Sci. USA.

[35] Li L., Zhang H., Chen C., Huang H., Tan X., Wei Z., Li J., Yan F., Zhang C., Chen J. (2021). A class of independently evolved transcriptional repressors in plant RNA viruses facilitates viral infection and vector feeding. Proc. Natl. Acad. Sci. USA.

[36] Sun Z., He Y., Li J., Wang X., Chen J. (2014). Genome-Wide Characterization of Rice Black Streaked Dwarf Virus-Responsive MicroRNAs in Rice Leaves and Roots by Small RNA and Degradome Sequencing. Plant Cell Physiol..

[37] Livak K.J., Schmittgen T.D. (2001). Analysis of Relative Gene Expression Data Using Real-Time Quantitative PCR and the 2^−ΔΔCT^ Method. Methods.

[38] Seifert E. (2014). OriginPro 9.1: Scientific Data Analysis and Graphing Software—Software Review. J. Chem. Inf. Model..

[39] Toriyama S., Takahashi M., Sano Y., Shimizu T., Ishihama A. (1994). Nucleotide sequence of RNA 1, the largest genomic segment of rice stripe virus, the prototype of the tenuiviruses. J. Gen. Virol..

[40] Du Z., Xiao D., Wu J., Jia D., Yuan Z., Liu Y., Hu L., Han Z., Wei T., Lin Q. (2011). p2 of Rice stripe virus (RSV) interacts with OsSGS3 and is a silencing suppressor. Mol. Plant Pathol..

[41] Wei T.-Y., Yang J.-G., Liao F.-L., Gao F.-L., Lu L.-M., Zhang X.-T., Li F., Wu Z.-J., Lin Q.-Y., Xie L.-H. (2009). Genetic diversity and population structure of rice stripe virus in China. J. Gen. Virol..

[42] Xiao D., Li W., Wei T., Wu Z., Xie L. (2010). Advances in the studies of Rice stripe virus. Front. Agric. China.

[43] Wei T., Li Y. (2016). Rice Reoviruses in Insect Vectors. Annu. Rev. Phytopathol..

[44] Zhao S.-P., Xu Z.-S., Zheng W.-J., Zhao W., Wang Y.-X., Yu T.-F., Chen M., Zhou Y.-B., Min D.-H., Ma Y.-Z. (2017). Genome-Wide Analysis of the RAV Family in Soybean and Functional Identification of *GmRAV-03* Involvement in Salt and Drought Stresses and Exogenous ABA Treatment. Front. Plant Sci..

[45] Lin H., Zhu W., Silva J.C., Gu X., Buell C.R. (2006). Intron gain and loss in segmentally duplicated genes in rice. Genome Biol..

[46] Rose A.B., Reddy A.S.N., Golovkin M. (2008). Intron-Mediated Regulation of Gene Expression. Nuclear Pre-mRNA Processing in Plants.

[47] Brenchley R., Spannagl M., Pfeifer M., Barker G.L.A., D’Amore R., Allen A.M., McKenzie N., Kramer M., Kerhornou A., Bolser D. (2012). Analysis of the bread wheat genome using whole-genome shotgun sequencing. Nat. Cell Biol..

