# Supplementary material for: Genome-Wide Analysis of the RAV Transcription Factor Genes in Rice Reveals Their Response Patterns to Hormones and Virus Infection
Source: Viruses. 2021 Apr 25;13(5):752. doi: 10.3390/v13050752 (PMC8146320; doi:10.3390/v13050752)
Supplement: Supplementary file 1 [file viruses-13-00752-s001.zip › viruses-1178948-supplementary.pdf]

Table S1. List of primers used for relative expression level

Gene	Primer	Primer sequence
OsUBQ	RT-UBQ5-2F	ACCACTTCGACCGCCACTACT
	RT-UBQ5-2R	ACGCCTAAGCCTGCTGGTT
OsRAV1	RT-OsRAV1-F	CATGTTCGAGAAGGTGGTGAC
	RT-OsRAV1-R	CCTTGGTGATGACGTAGCTCT
OsRAV2	RT-OsRAV2-F	GCACATGTTTGAGAAGGTCGT
	RT-OsRAV2-R	TTGGTCATCACGTAGCTCTGG
OsRAV3	RT-OsRAV3-F	CACATGTTCGACAAGGTGGT
	RT-OsRAV3-R	TGGTCATCACGTAGCTCTGG
OsRAV4	RT-OsRAV4-F	ACATGTTCGACAAGGTGGTGA
	RT-OsRAV4-R	TCGAGTCGCTTCTCCTTGAC
OsRAV5	RT-OsRAV5-F	CGACTTCCGCAACATCAACC
	RT-OsRAV5-R	GGTGCACCGTAATGTGGAGT
OsRAV6	RT-OsRAV6-F	AGGCGAGAGATCCCCTTCAT
	RT-OsRAV6-R	ACTTCTCGGCGTACTGCTTC
OsRAV7	RT-OsRAV7-F	ATGATCACGGGGACATGCAC
	RT-OsRAV7-R	GCAGACATGGCAGCATAGGA
OsRAV8	RT-OsRAV8-F	AGCCGATTCGTGAGGGAGAA
	RT-OsRAV8-R	TCTTGTCTCCGGCGATGTCA
OsRAV9	RT-OsRAV9-F	CGGCGACACCATAGTCTTCT
	RT-OsRAV9-R	CGCTGATGAACCACGCATTG
OsRAV10	RT-OsRAV10-F	AGCACGTCGGGAAACTACTG
	RT-OsRAV10-R	CGTGAATGCGATGCGATGTT
OsRAV11	RT-OsRAV11-F	TCCGCAAGCACACCTACTTC
	RT-OsRAV11-R	TGCTGCTTCGGTATGACGAG
OsRAV12	RT-OsRAV12-F	GACGTCGGGAAGCTGAACC
	RT-OsRAV12-R	GCTGTTCCAGTAGGAGTACCG
OsRAV13	RT-OsRAV13-F	CAGAGCTACGTGCTCACCAA
	RT-OsRAV13-R	GGCATATGAATTGGCAGGGC
OsRAV14	RT-OsRAV14-F	TGTTGGATTTGTCGGGCTGT
	RT-OsRAV14-R	AGGCAGTAAACAGGCAGGTC
OsRAV15	RT-OsRAV15-F	TGGTGGGTCTTCAATCTGGC
	RT-OsRAV15-R	TTGGTGGCGTCTCTATCGTG

Table S2. Properties of the maize RAV proteins identified, including sequence ID and predicted sequence length.

number	Gene ID number	Amino acid residues	B3 domain	AP2 domain
1	Zm00001d039907_T001	378	186-282	74–130
2	Zm00001d035903_T001	224	26-121	
3	Zm00001d043782_T001	389	213-330	77-134
4	Zm00001d024103_T001	224	26-121	
5	Zm00001d023446_T001	283	41-133	
6	Zm00001d010077_T001	259	40-125	
7	Zm00001d047359_T001	277	32-128	
8	Zm00001d011639_T001	405	212-308	81-137
9	Zm00001d051471_T001	420	84-173	
10	Zm00001d029749_T001	273	30-127	
11	Zm00001d048815_T001	232	40-122	
12	Zm00001d052591_T001	307	43-140	
13	Zm00001d017618_T001	422	101-188	
14	Zm00001d038907_T001	406	206-313	73-134
15	Zm00001d052591_T002	305	43-140	

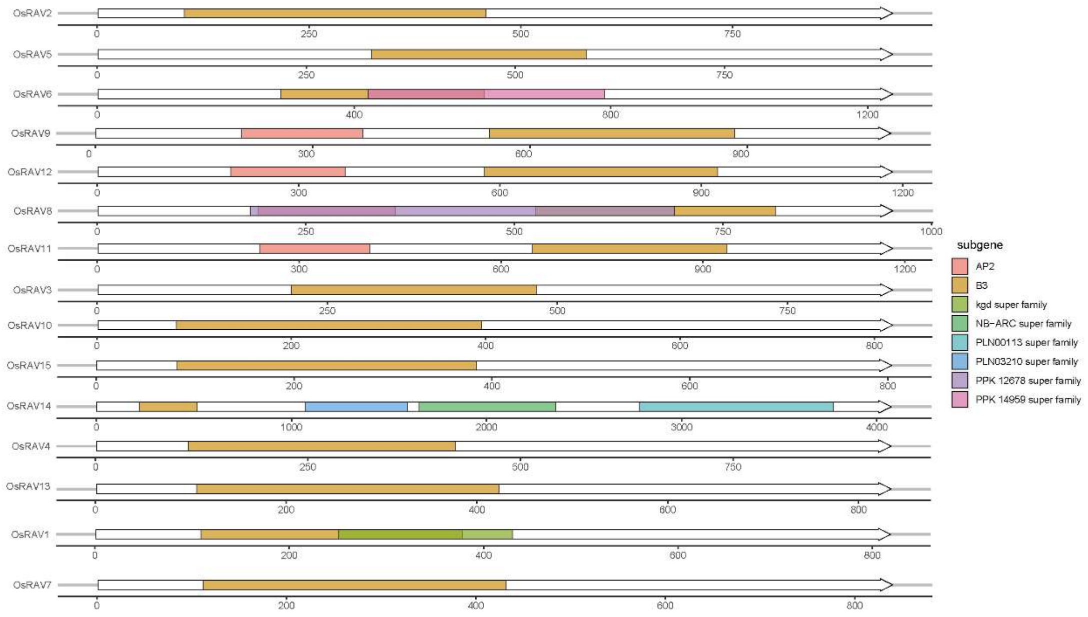

Figure S1 Architecture of conserved protein motifs in RAV genes from rice. The AP2 domains were highlighted by pink boxes and the B3 domain by light yellow boxes. The motifs, numbers 3–8, were displayed in different colored boxes. The sequence information for each motif is provided in the attached file. Protein and gene length can be estimated using the bottom ratio.
